# Thyroid Tissue Connected to Normally Located Thyroid Gland: Ectopic or Exophytic?

**DOI:** 10.1155/2012/681823

**Published:** 2012-10-09

**Authors:** Erol Keles, Sule Ozkara, Turgut Karlidag, İbrahim Hanifi Ozercan

**Affiliations:** ^1^ENT Department, Firat University, Elazig, Turkey; ^2^Pathology Department, Firat University, Elazig, Turkey

## Abstract

Ectopic thyroid tissue is seen rarely. It is often seen in cervical midline, and rarely in other areas such as submandibular area. Diagnosis is made histopathologically by fine needle biopsy after the elimination of malignancy. In the treatment of ectopic thyroid tissue, surgical excision is mostly applied. According to our knowledge, there is no exophytic thyroid tissue reported in the literature. In this paper, a 32-year-old woman who presented with a swelling under the right jaw and found a thyroid tissue attached to the normally located thyroid gland with a fibrous band in the neck was discussed.

## 1. Background

Embryologically, thyroid tissue develops with the fusion of medial and lateral cell clusters [1]. Lateral pole forms 1–30% of total thyroid weight and develops from fourth pharyngeal pouch. Medial pole is the largest part of the thyroid parenchyma [2]. Various anomalies such as ectopic thyroid tissue may occur during the development of the thyroid gland. Ectopic thyroid tissue may occur either with or without the normal thyroid gland [2]. The problems in downward development of the medial part conclude with the lingual thyroid development [1]. In rare cases, fusion deficiency of the medial and lateral cell clusters that generate the thyroid causes to form the lateral ectopic thyroid tissue. It concludes with the development of thyroid tissue located in submandibular area. In literature, there are almost 25 published ectopic thyroid cases which were only submandibular located without any functional ectopic thyroid tissue.

The tissue growing outside toward surface of the organ it was originated from or another structure is defined as exophytic tissue. These tissues are in connection with a structure that has similar functional features with the organ it is derived from. We did not find any exophytic thyroid tissue based on the literature review.

In this paper, a 32-year-old woman who was diagnosed with a thyroid tissue attached to the normally located right thyroid gland lobe with a fibrous band in the neck was presented.

## 2. Case Presentation

A 32-year-old woman presented with a 4-year history of swelling under the right jaw. Neck USG showed a hypoechoic heterogeneous solid nodular lesion in size of 22 × 15 mm, also consisting of cystic components, located exophytically in the right submandibular area, next to the right thyroid lobe upper pole. The fine needle aspiration biopsy from the submandibular region showed thyrocyte groups in which some has narrow cytoplasm with nuclear cleavage and forming clustered microfollicles, and pathology was reported as insignificant atypia.

**Figure 1:**
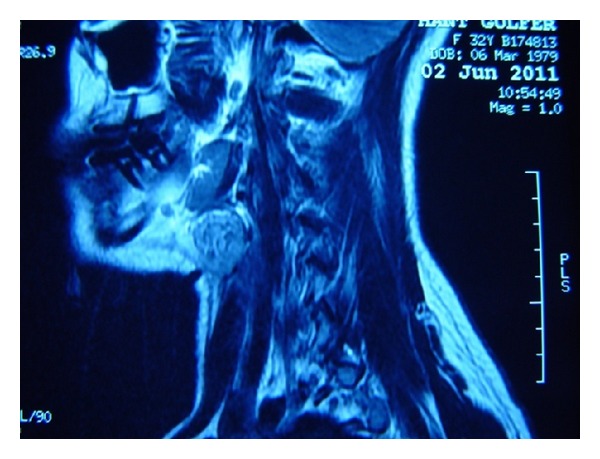
Neck MRI with contrast (sagittal view); nodular lesion with sharp contours, originated exophytically at right thyroid lobe upper pole, located in right level II, without an invasion to the nearby strap muscles.

**Figure 2:**
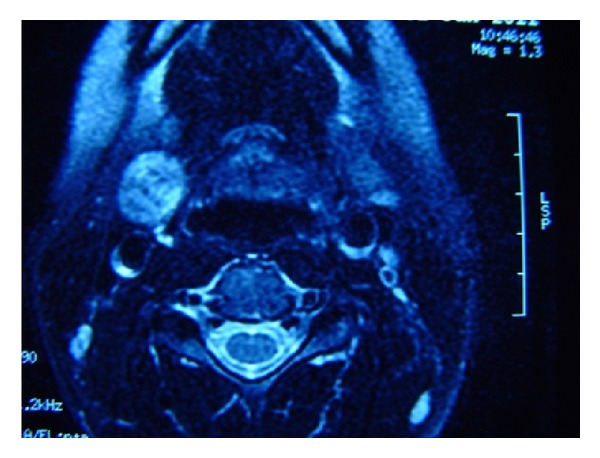
Neck MRI with contrast (axial view); hyperintense, heterogeneous nodular lesion with sharp contours, located in right level II and anterior of carotid sheath, pushes the vascular forms to the posterior, without an invasion to the nearby strap muscles.

Neck MRI with contrast detected a mass in size of 17 × 17 × 23 mm with sharp contours, located in right level II, minimally hyperintense compared to nearby muscles in T1A series, significantly hyperintense in T2A series, and without suppression in fat-suppression sequences. The mass was considered as a nodular lesion mostly in right thyroid lobe upper part, originated exophytically, without an invasion to the nearby strap muscles (Figures 1 and 2).

In preoperative assessment of the patient; hemogram, biochemistry, and thyroid function tests were in the normal range. With these findings, surgical excision was planned. Mass excision was done along with the lobectomy and isthmusectomy. Intraoperatively, it was detected that the submandibular mass was connected to the right thyroid lobe superior medial part with a fibrous band. Perioperatively, frozen section result was reported as benign.

**Figure 3:**
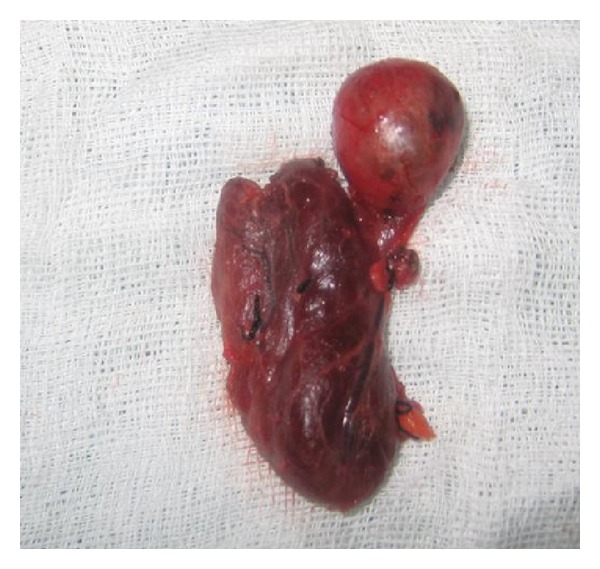
Macroscopic view of the mass.

**Figure 4:**
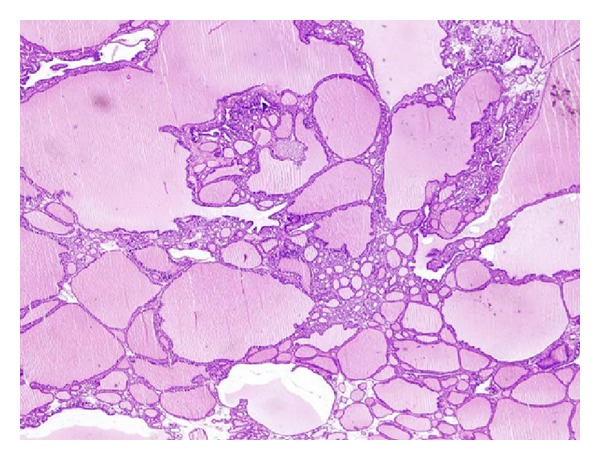
Microscopic view of the specimen.

Pathological review of the specimen demonstrated that the right submandibular mass was a thyroid tissue in size of 2, 2 × 1, 7 × 1, 2 cm attached to a thyroid tissue in size of 6,5 × 3 × 1, 5 cm with a fibrous stalk (Figures 3 and 4). No recurrences was seen at 9-month postoperative followup, and no abnormalities were detected in thyroid function tests.

## 3. Discussion

Thyroid tissue, which is located outside of the anterolateral second, third, and fourth tracheal rings, is defined as ectopic thyroid tissue and it presents the actual form of the thyroid dysgenesis. Ectopic thyroid gland occurs 1 in 4,000 to 8,000 patients with the thyroid diseases and male to female ratio is 1/4 [3]. First ectopic thyroid case was reported in a newborn presented with an upper airway obstruction 16 hours after birth as a lingual thyroid by Hickman in the year of 1869. Ectopic thyroid tissue is mostly in midline between foramen cecum and mediastinum and rarely may locate laterally after median migration disorders. Almost 90% of case reports state lingual location. Submandibular location of the thyroid tissue is very uncommon [2].

While 90% of ectopic thyroid tissues are located midline, 10% may locate in a different anatomic area [1]. Althoughan ectopic thyroid tissue in submandibular area is extremely rare, it should be considered in differential diagnosis of the neck masses. Ectopic thyroid tissue is usually in midline of the neck as lingual, sublingual, prelaryngeal, and intralaryngeal. Nonetheless, nonmidline ectopic thyroid tissues which are located submandibular, in the wall of aorta, intracardiac, and in liver have been described [4–6].

The tissue growing outside toward the organ surface it was originated or another structure is defined as exophytic tissue. These tissues are in connection with a structure that has similar functional features with the organ it is derived from [7]. We did not find any exophytic thyroid tissue based on the literature review.

 In our case, submandibular located thyroid tissue was attached to the normally located thyroid tissue (right thyroid lobe) with a fibrous band, which does not contain a functional thyroid tissue. This fibrous attachment was not defined as exophytic since it did not contain any thyroid tissue. Ectopic tissues are the structures that do not connect to the normally located tissues in an area other than it is supposed to be located. In our case, since submandibular located thyroid tissue was connected to the normally located thyroid tissue, it could not be defined as ectopic tissue, either.

As this clinical case is uncommon, there is no consensus on optimal treatment method. Many researchers state that the surgical treatment of the ectopic thyroid located in the neck (lingual, sublingual, submandibular, and lateral cervical) is related to the mass size and local symptoms (airway obstruction, dysphagia, and dysphonia). While deciding to the surgical treatment, the factors such as patient age, functional thyroid tissue, and the complications of the mass such as ulceration, bleeding, or malignancy are also crucial [8].

We consider to present this case since we had difficulty to name this situation. We believe that surgical resection and pathological assessment are the optimal treatment methods since these lesions carry a risk of hidden thyroid cancer metastasis or primary cancer.
